# Design, manufacturing and experimental validation of additive manufacturing cores for bearing seats in carbon fibre reinforced polymer structures

**DOI:** 10.1016/j.heliyon.2024.e35652

**Published:** 2024-08-05

**Authors:** Guillermo Retuerta del Rey, Javier de Lucas Salgado, Alex Alberto González Hernández, Enrique Chacón Tanarro

**Affiliations:** a Grupo de Investigación en Ingeniería de Fabricación, Departamento Ingeniería Mecánica, Universidad Politécnica de Madrid, Spain; b GI-IM, Departamento Ingeniería Mecánica, Universidad Politécnica de Madrid, Spain

**Keywords:** Carbon fibre reinforced polymers (CFRP), Additive manufacturing enabled composites, Mechanical testing, Polymer inserts

## Abstract

The integration of carbon-fibre reinforced polymers (CFRP) in structural applications offers significant advantages due to their high strength-to-weight ratio. However, these materials exhibit limitations under out-of-plane loads, particularly in bearing applications. This study explores an innovative approach to enhance the performance of CFRP structures in such scenarios by incorporating annular polyamide inserts manufactured via additive manufacturing (AM).

To evaluate the mechanical performance of the AM inserts, a novel ring tensile test is designed to emulate the bearing load conditions. This test also enables the analysis of the impact of several design and manufacturing parameters of the AM inserts, including surface geometry, surface treatment, internal structure, and curing process. These are then compared with specimens made of carbon fibre sheet moulding compounds (CF-SMC), commonly used to support bearing loads. The study reveals that AM inserts provide a viable alternative to state-of-the-art CF-SMC, offering a significant enhancement in mechanical properties under specific bearing loading conditions. The test results indicate a 17.7% improvement in the first failure limit and an 8.6% increase in the ultimate strength for AM inserts compared to CF-SMC.

Additionally, the study develops a simplified analytical model to predict stress distributions and potential failure mechanisms, validating its efficacy through experimental data with discrepancies of less than 6%. Economic analysis underscores the cost benefits of AM inserts due to reduced labour and higher repeatability. This research demonstrates the potential of AM inserts to improve the stability and strength of CFRP structures, paving the way for their broader application in demanding load-bearing environments.

## Highlights

• Introduction of additive manufacturing inserts in CFRP laminates for bearing seats.
• Development of ring test methodology for composite bearing seats.
• Comparison of AM inserts against state-of-the-art SMC designs.

## 1 Introduction

Composite materials are extensively utilized across various sectors, including aerospace, automotive, and sports goods, due to their ability to combine the distinct properties of the materials they are composed of. Among these, continuous Carbon Fibre Reinforced Polymers (CFRP) are notable for their exceptional strength-to-weight ratio, particularly when loads align with fibre directions. However, continuous CFRP laminates exhibit limited performance under out-of-plane loading conditions, as numerous studies demonstrate [[1], [2], [3]]. Additionally, continuous CFRP structures are often designed as thin sections to optimise weight and fully leverage the material's capabilities. Such thin structures, however, are inadequate for withstanding flexural loads. In certain CFRP structures, out-of-plane and flexural loads are inevitable in local areas. Examples include aircraft wing spars [4], suspension arms' bushings [5], and bicycle steering tubes' bearing seats. Consequently, manufacturing CFRP components capable of enduring these loading conditions requires the exploration and implementation of alternative methodologies.

**Fig. 1:**
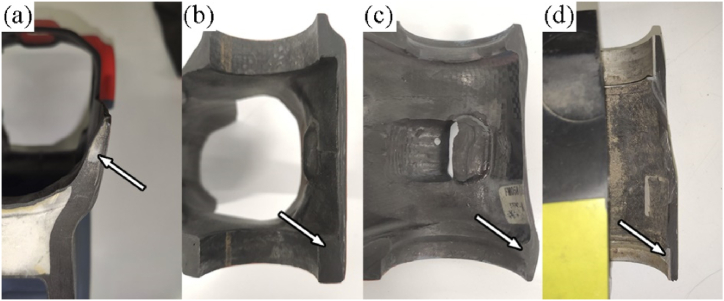
Cuts made in bicycle frames. The bearing load reinforcements are made with foams (a), continuous CFRP (b), CF-SMC (c), and metal inserts (d). The arrows point to the reinforcement element in each structure.

To address these limitations, various solutions have been developed and employed in recent years. Foam cores, as shown in Fig. 1a, are used due to their extreme lightness and reduced costs [6]. However, their reduced mechanical properties make them unsuitable for certain high-demand applications. Alternatively, CFRP-based solutions are used to enhance strength, as shown in Fig. 1b and c. Of particular interest is the use of Carbon Fibre Sheet Moulding Compounds (CF-SMC), as employed by Lamborghini in the manufacturing of suspension arms [7]. This material is suitable for withstanding the stresses transferred by bushings due to its formability, despite exhibiting a reduction of 65% in tension and compression strength and a 22% reduction in bending strength when comparing to continuous CFRP [8]. CF-SMC is also used by L.M. Martulli et al. in the construction of thick-walled automotive components [9], where it is concluded that due to intrinsic manufacturing variability, no significant effect of any process parameters can be observed. This variability is attributed to the intricate flow pattern produced during the compression moulding process, resulting in a random distribution of fibre bundles and unpredictable mechanical properties [10]. Therefore, the uncertainty in the performance of these structures necessitates alternative solutions.

To mitigate the uncertainties associated with CF-SMC solutions, metallic inserts are used as cores for composite structures. J. Xiong et al. study the integration of a glass fibre core and a metallic inner ring in tensile joints for a CFRP rapid portable bridge [11]. D.A. Türk et al. incorporate a load introduction element, fabricated from titanium via additive manufacturing, in biomedical and mobile robotics applications [12,13]. These approaches address the manufacturing variability challenges present in CF-SMC. However, their use is limited due to corrosion concerns, as explained by G. Song et al. [14], and their elevated weight compared to other alternatives.

A potential alternative to the mentioned manufacturing processes is the integration of a polymer-made annular insert via additive manufacturing (AM) within the continuous CFRP laminate. This approach leverages the main advantages of the previously mentioned techniques. AM processes ensure full automation in manufacturing the inserts, thus guaranteeing repeatability in their mechanical properties. Furthermore, by selecting appropriate materials and manufacturing parameters, strength-to-weight ratios comparable to CF-SMC may be achieved. Material extrusion of polymers (MEX/P) is the most widespread AM technology due to its cost-effectiveness and the simplicity of the required equipment and materials [15]. This manufacturing technique allows the orientation of filament direction to align optimally with stress distributions [16,17], optimising the mechanical behaviour of the insert. A widely used material in MEX/P is polyamide (PA). PA filaments emerge as a suitable material for fabricating AM inserts that must endure an epoxy curing process, given their thermal properties and bonding compatibility [[18], [19], [20], [21]].

In this context, the implementation of a unified testing methodology for comparison with other manufacturing alternatives is necessary. Bearing seats serve as a representative load case for out-of-plane loads. The use of annular inserts in bearing seats highlights the need for a suitable mechanical test to simulate these working conditions. Among the observed alternatives [[19], [20], [21], [22], [23], [24]], ring tensile tests are the most appropriate for replicating the load distribution of a bearing seat.

This paper demonstrates the feasibility of using additive manufacturing inserts as cores for carbon fibre reinforced polymer structures to withstand bearing loads. This innovative approach provides a viable alternative to the current state-of-the-art carbon fibre sheet moulding compound structures or metallic inserts, offering an optimal combination of lightness, strength, and repeatability.

This work proposes a unified testing methodology for CFRP rings. This methodology enables the comparison of AM inserts with CF-SMC solutions. Various design and manufacturing parameters of the inserts are studied to understand their influence on the mechanical properties of the assembly and to identify the optimal combination. A simplified linear model is developed to design the test specimens and the test itself, serving as a predictive tool for the behaviour of future similar designs.

## 2 Methodology

To verify the proposed hypotheses in this work, the presented methodology begins with the development of an analytical model that not only allows for the calculation of internal stresses based on the experimental results but also facilitates the design of the mechanical test and the specimens. Following this theoretical structural analysis, subsequent subsections provide detailed descriptions of the specimens, including their geometry, materials, and process parameters. Later, the mechanical testing methodology is presented and finally, a test plan is presented.

### 2.1 Theoretical structural analysis

**Fig. 2:**
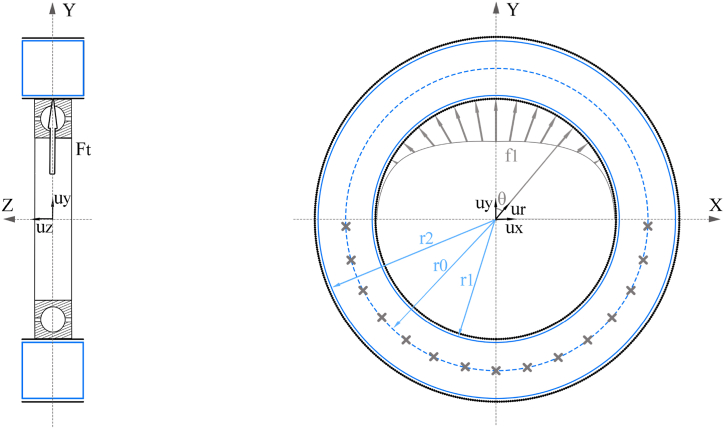
Schematic representation of a generic bearing seat subjected to a radial bearing load. CFRP laminates are represented in black, reinforcement material in blue and load conditions in grey. (For interpretation of the references to colour in this figure legend, the reader is referred to the Web version of this article.)

To ensure the efficacy of the designed structure in real-world applications, it is crucial to test it under conditions that replicate its intended use. This study considers the loads applied to the bearing seat structure. The bearing seat structure, as shown in Fig. 2, is simplified into a constant section ring. A bearing load is applied to one half of its inner face, while the other half is fixedly supported. A mathematical linear model is developed to comprehend the stress distribution and identify the critical areas where cracks may originate. In Fig. 2, the crosses represent the fixed supports, f_1_ is the force per area unit which represents the bearing load distribution, r_1_ is the inner radius of the ring, r_2_ is the outer radius, and r_0_ is the mean radius of the ring specimen which is defined as (1):

(1) $$r_{0}=\frac{\left(r_{1}+r_{2}\right)}{2}$$

Given $F_{t}$ as the resultant of the force applied along the y axis, $f_{\max}$ as the maximum value of the load distribution, and l as the thickness of the insert along the z axis, force distribution $f_{1}$ is defined by the expression of a sinusoidal distribution [22]:

(2) $$\vec{f_{1}}=f_{\max}\cdot \cos\;\theta \;\vec{u_{r}}$$

(3) fmax=Ft2l·r1·π4

**Fig. 3:**
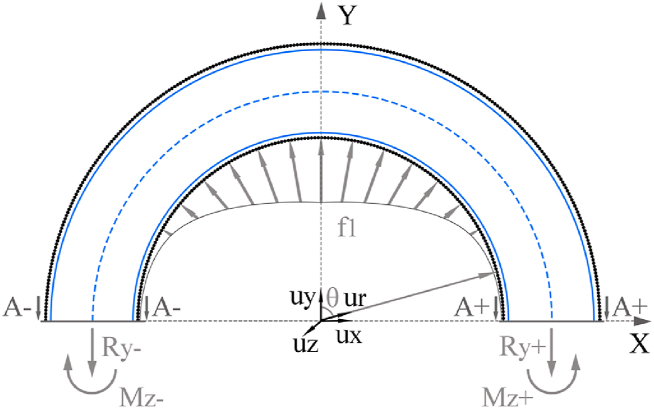
Schematic representation of half of the steering tube structure, the bearing load distribution and internal stresses in A- and A+ cut sections.

The test specimen comprises a core constructed from the reinforcement material under analysis, with CFRP laminates on its internal and external surface. The stress supported by each section is computed as the sum of tensile and flexural stress distributions induced by $f_{1}$. Sections located in the x axis in Fig. 2 experience the highest flexural stresses as they are the ones located farthest from the $f_{1}$ load distribution. Therefore, they are considered the critical operation sections where breaks are originated. To understand the stress conditions that these sections must support, a cut view of the upper half of the structure is shown in Fig. 3, where critical operation sections are called A- and A+. $M_{z-}$ and $M_{z+}$ are the flexural moments along the out of plane axis, and $R_{y-}$ and $R_{y+}$ are the normal reaction forces along the y axis for sections A- and A+, respectively. Considering the symmetry of the model in both geometry and load distribution, the equilibrium of forces and flexural moment results in the following expressions:

(4) $$\vec{R_{y+}}=\vec{R_{y-}}=\frac{F_{t}}{2}\vec{u_{y}}$$

(5) $$\vec{M_{z+}}=-\vec{M_{z-}}$$

**Fig. 4:**
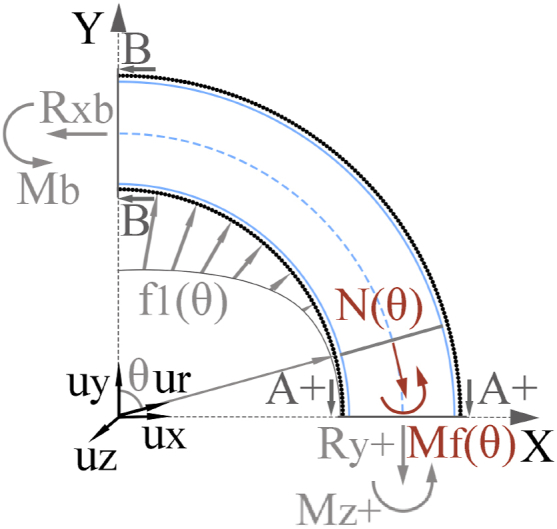
Schematic representation of a quarter of the bearing seat structure, the bearing load distribution, and internal stresses in A+ and B cut sections.

Since Fig. 3 represents a hyperstatic structure, the expressions for $M_{z-}$ and $M_{z+}$ cannot be found from a moment equilibrium. To calculate these values, a quarter of the structure must be considered, as represented in Fig. 4. Section B is generated after cutting Fig. 3 through the y axis. $R_{xb}$ represents a reaction force in the x axis direction in section B, which opposes the x component of the bearing load:

(6) Rxb→=∫0π2fmax·l·r1·sinθ·cosθ·dθ·ux→

$M_{b}$ represents the flexural moment supported by section B. Therefore, the expression of $M_{z+}$ is as follows:

(7) $$M_{z+}={-M}_{b}-r_{0}{\cdot R}_{xb}+r_{0}\cdot \frac{F_{t}}{2}$$

The expression of the normal reaction force at a section defined by angle θ is:

(8) $$N\left(\theta \right)=\cos\left(\theta \right)\cdot R_{\text{xb}}+\frac{F_{t}}{2}\cdot \frac{2}{\pi }\cdot \theta \cdot \sin\left(\theta \right)$$

The expression of the flexural moment at the same section is:

(9) $$M_{f}\left(\theta \right)={-M}_{b}-r_{0}{\cdot \left(1-\cos\left(\theta \right)\right)\cdot R}_{\text{xb}}+r_{0}\cdot \frac{F_{t}}{2}\cdot \frac{2}{\pi }\cdot \theta \cdot \sin\;\left(\theta \right)$$

Considering eq. (9), the moment $M_{b}$ must be obtained prior to knowing $M_{z+}$. This moment can be obtained by minimizing the strain energy of this model, as it is resolved in other works [23,24]. Thicker rings, however, need to consider more terms of strain energy [25]. Therefore, before the size of the ring is determined, the model is set to be a thick section ring for the sake of generality. The model's total energy terms which depend on $M_{b}$ are the following:

(10) $$U_{Mb}=\int \frac{{M_{f}}^{2}}{2\cdot E\cdot I_{z}}\cdot ds-\int \frac{M_{f}\cdot N}{A\cdot E\cdot r_{0}}\cdot ds$$

Since the ring does not rotate during the test, Castigliano theorem determines that [25]:

(11) $$\frac{dU_{Mb}}{dM_{b}}=0$$

**Fig. 5:**
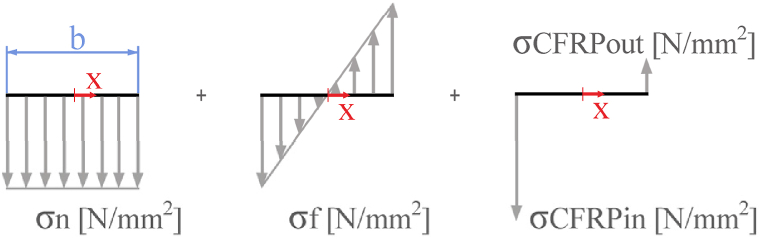
Deconstructed stress distribution in A+ section. On the left, the tensile load distribution in the insert. Section length is defined as s. In the centre, the flexural stress distribution in the insert. On the right, the tensile stresses in CFRP laminates.

From Castigliano theorem, the moment M_b_ can be calculated through the following expression:

(12) Mb=r0·Rxb·2π−1+r0·Ft2π24−2·IzA·r0·π·Rxb+Ft2π4

with the expression of the flexural moment in section A+, it is possible to obtain the stress distribution in the reinforcement material and in the CFRP laminates. The core of reinforcement material must sustain a distribution of flexo-tensile stresses, as it is represented in Fig. 5:

(13) $$\sigma_{Core}\left(x\right)=\sigma_{n}\left(x\right)+\sigma_{f}\left(x\right)$$

$\sigma_{Core}\left(x\right)$ denotes the sum of the stress generated by both flexural and tensile effects. The tensile stress component is concluded to be constant through the section. Taking the reference system of Fig. 5, and denoting $\sigma_{n}\left(x\right)$ as the tensile stress in N/mm^2^ and q as the constant term of tensile stress distribution in N/mm^2^, the expression of tensile stress in the core material is as follows:

(14) $$\sigma_{n}\left(x\right)=q$$

The flexural stress component varies throughout the section as a result of moment $M_{z+}$. Denoting $\sigma_{f}$ as the flexural stress in N/mm^2^, k as the constant term of flexural stress distribution in N/mm^3^, and referring the x coordinate in mm, the expression of flexure stress in the core material is determined by:

(15) $$\sigma_{f}\left(x\right)=k\cdot x$$

CFRP laminates in the inner and outer surfaces resist higher tensile stress than the core material due to its higher stiffness. Denoting $\sigma_{CFRPin}$ as the tensile stress in the inner laminate in N/mm^2^, $\sigma_{CFRPout}$ as the tensile stress in the outer laminate in N/mm^2^, $R_{in}$ as the total load in the inner laminate in N, $R_{out}$ as the total load in the outer laminate in N, and t as the laminates thickness in mm, the expressions of tensile stresses in both CFRP laminates are the following:

(16) $$\sigma_{CFRPin}=\frac{R_{in}}{l\cdot t}$$

(17) $$\sigma_{CFRPout}=\frac{R_{out}}{l\cdot t}$$

The core material undergoes deformation identical to that of the adjacent CFRP laminates. Equating the tensile stresses with the load in the section, balancing the moments at the origin of the section, and equating the deformation expressions of linear materials at both ends of the section yields a compatible system of four equations. Denoting b as the length of the section as represented in Fig. 5, $E_{Core}$ as the Young's modulus of the core material, and $E_{CFRP}$ as the Young's modulus of the CFRP laminates in the fibres direction, the following system is obtained:

(18) ∫−b2b2q·l·dx+R1+R2=Ry+

(19) ∫−b2b2k·x2·l·dx−b2·R1+b2·R2=Mz+

(20) R1l·t·ECFRP=q+k·-b2ECore

(21) R2l·t·ECFRP=q+k·b2ECore

The bond between the reinforcement material and the CFRP laminates suffers a shear strength equal to the difference in stresses between the two materials. Denoting $\tau_{in}$ and $\tau_{out}$ as the shear stresses developed in the bond at the inner and outer interfaces, respectively, the following expressions are obtained:

(22) τin=σCFRPin−σCore(−b2)

(23) τout=σCFRPout−σCore(b2)

**Fig. 6:**
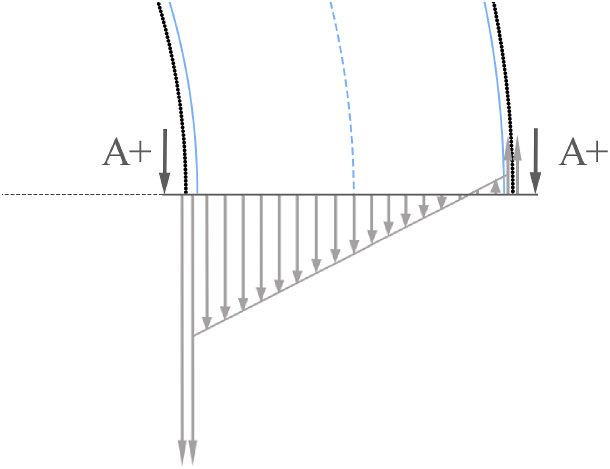
Amplified schematic representation of the internal stresses withstood by the bearing seat structure. CFRP laminates are represented in black, reinforcement material in blue and load conditions in grey. (For interpretation of the references to colour in this figure legend, the reader is referred to the Web version of this article.)

Fig. 6 provides a more detailed representation of the stress distribution in the A- and A+ sections. In this representation, it is considered that the CFRP laminates have higher stiffness than the reinforcement material, resulting in $E_{Core}<E_{CFRP}$. The highest stresses are located on the inner face of the ring.

The mathematical linear model, in conjunction with the schematic representation in Fig. 6, predicts the failure of the structure to originate in one of three ways at the inner points of sections A- and A+. The first potential origin is the CFRP laminates, which can withstand higher stress levels than reinforcement materials, but also bear the maximum stress in the model. The second possible origin is the reinforcement material itself, which may fail upon reaching its break limit, which is influenced by its internal structures [26]. Lastly, the discrepancy in stresses between the reinforcement material and CFRP laminates can generate a shear stress, potentially leading to delamination. To enhance the specimen's interface strength, surface treatments such as macroscopic geometric patterns [[27], [28], [29], [30], [31]] or sandblasting treatments [13] can be employed to augment mechanical interlocking.

### 2.2 Specimens geometry

**Fig. 7:**
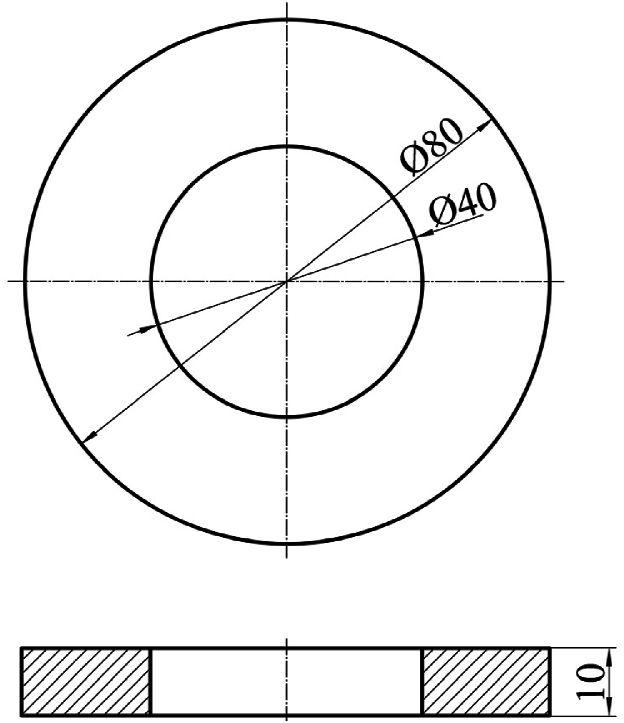
Schematic representation of the final geometry of the inserts.

In this study, the specimens are designed to emulate the behaviour of bearing seat structures, possessing an annular geometry as depicted in Fig. 7. To emulate the integration of inserts between CFRP laminates, these specimens are characterized by the bonding of prepreg sheets to both their outer and inner surfaces. This design choice ensures a more accurate representation of the real-world conditions these materials would encounter.

**Fig. 8:**
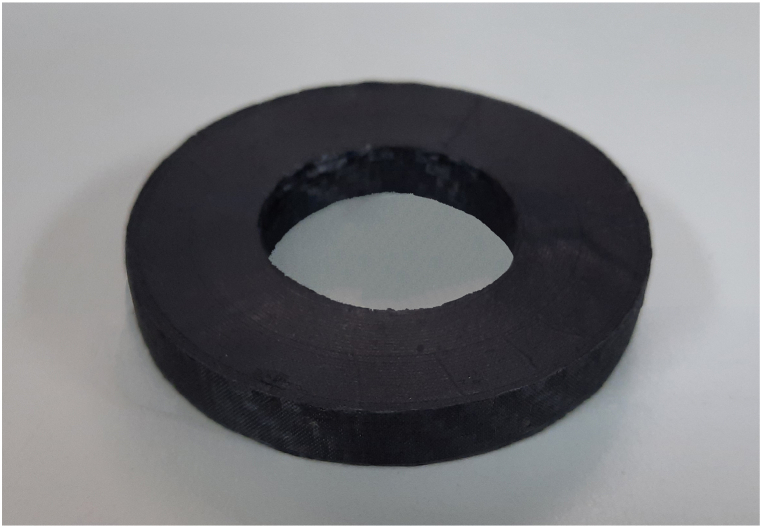
AM insert after CFRP curing process. Prepreg sheets are bonded on the exterior and interior cylindrical surface of the ring specimen.

As illustrated in Fig. 7, the specimens are characterized by specific dimensions. The inner and outer diameters measure 40 mm and 80 mm, respectively, while the height is fixed at 10 mm. This configuration yields a critical operation section with dimensions of 20 × 10mm. The selection of these dimensions aims to minimize the variability effects attributable to both the manual lamination of the prepreg layers and the inherent variability of the mechanical properties of AM inserts and CF-SMC. The thickness of the CFRP laminates is significantly less than the size of the section, measuring 0.33 mm. The manufactured specimen is presented in Fig. 8 for further reference.

### 2.3 Materials

State-of-the-art specimens manufactured with CF-SMC cores are fabricated as reference for comparison with the novel alternative composed of CFRP laminates with AM cores. The analysis of both materials is presented in this section.

#### 2.3.1 CFRP

**Fig. 9:**
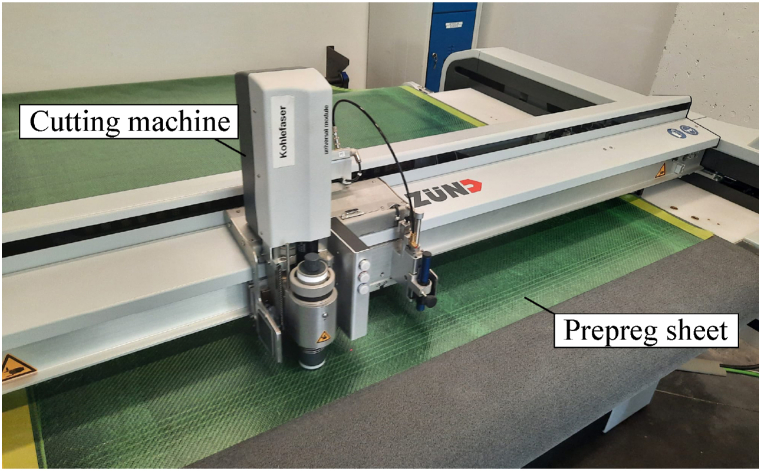
Cutting process of CFRP prepreg layers. Prepreg layers are covered with protective green film. (For interpretation of the references to colour in this figure legend, the reader is referred to the Web version of this article.)

In both specimen types, prepreg layers of CC206 ET445 43% T300 3k, provided by CIT, are utilized to emulate the integration of the inserts into bearing seats. The most relevant properties of this material are detailed in Table 1. Following a curing cycle at 125 °C and 3 bar for 30 min, without a subsequent post-cure process, the composite material achieves a tensile strength of 634 MPa, a compressive strength of 836 MPa, a Young's modulus ($E_{CFRP}$) of 53.5 GPa under tensile conditions and 44.9 GPa under compressive conditions, aligned with the fibre direction. The cutting process of the prepreg layers is depicted in Fig. 9.

**Table 1:** Properties of prepreg sheets CC206 ET445 43% T300 3k provided by the manufacturer.

**Dry fabric**		**Uncured prepreg**		**Cured properties**	
Weaving style	Twill 2/2/Batavia 2/2	Track	low - medium	Glass transition temperature	126 °C
Fibre type	T300 3k	Flow	15 (±5 %) %	Tensile strength	634 Mpa
Fibre density	1.76 g/cm^3^	Out life (23 °C)	30 days	Tensile modulus	53.5 Gpa
Wrap	5.1 threads/cm	Storage life (−18 °C)	12 months	Compression modulus	44.9 Gpa
Weft	5.1 threads/cm	Nominal area weight	358 g/m^2^	Compression strength	559 Mpa
Areal weight	204 (±4 %) g/m^2^	Nominal resin content	43 (±3) Wt %	Flexural modulus	51.6 Gpa
		Volatile content	<1 wt %	Flexural strength	836 Mpa
		Nominal width	1250 mm	Inter-laminar shear strength	76.4 Mpa

The reference inserts are fabricated in accordance with state-of-the-art processing methodologies. A CF-SMC charge is placed into the mould and co-cured alongside surface prepreg layers. When compared to structures composed of continuous prepreg sheets, there is a noticeable decrease in both tensile strength and Young's modulus ($E_{Core}$). This is attributed to the fact that carbon fibres exhibit optimal mechanical properties when they are continuously arranged and tensioned. However, establishing these values can be challenging due to the inherent variability in the manufacturing process, which is contingent upon the positioning of the prepreg sheets [9].

#### 2.3.2 Additive manufacturing inserts

The AM inserts are manufactured using an Ultimaker S5 MEX machine. This process is chosen for freedom of design and the wide range of materials available. This technology has its own limitations too. On the one hand, the layered extrusion process implies a low surface resolution of the printed part [32] as well as low dimensional tolerances, which are further intensified by warping and shrinkage [33]. On the other hand, parts manufactured with this process show a relevant anisotropy where the mechanical properties in the stacking direction show reduced values compared to the direction perpendicular to the stacking [34,35].

The selected polymer must possess the capacity to withstand the elevated temperatures associated with the epoxy curing process, which occurs at 125 °C. This is crucial as the insert is required to be positioned between the prepreg layers prior to this process. Furthermore, the formation of an adhesive bond between the insert and the epoxy resin necessitates the selection of a polymer that is compatible with epoxy in terms of adhesive bonding. PA is a polymer that fulfils both these prerequisites. PA filaments emerge as a suitable choice for fabricating AM inserts designed to endure the epoxy curing process. PA filaments exhibit superior thermal properties compared to other available alternatives. Various commercial variants of PA demonstrate a heat deflection temperature (HDT) at 4.5 bar that surpasses 125 °C [18]. Additionally, PA and epoxy prove to be compatible materials for adhesive bonding, attributed to the formation of covalent bonds between the amine and epoxide groups [19].

PA can be effectively utilized in conjunction with short carbon fibre reinforcements. The integration of a certain proportion of short fibres enhances both the strength and stiffness in tensile and flexural modes in any direction [20]. This augmentation in adherend stiffness culminates in a more evenly distributed shear stress, supported by the adhesive bond [21]. Consequently, the infusion of a percentage of short carbon fibres into the polyamide leads to a reduction in stresses at the bond interface, thereby increasing the strength of the specimen. The introduction of continuous carbon fibre reinforcements similarly alters the mechanical properties, emulating the trend observed with the use of short fibres [15]. However, this necessitates more intricate machine configurations.

The AM material utilized in this study is PAHT CF15, supplied by BASF, with its properties presented in Table 2. Assuming dry conditions for both manufacturing and testing, the manufacturer claims the HDT of the material to be 145 °C at 0.45 MPa and 91 °C at 1.8 MPa. The material exhibits a tensile strength of 103.2 MPa and a Young's modulus ($E_{Core}$) of 8.4 GPa under tensile conditions and 8.3 GPa under flexural conditions, specifically in the printing direction. Of note, this material demonstrates lower strength and stiffness compared to continuous prepregs laminates.

**Table 2:** Properties of AM filament PAHT CF15 in dry conditions provided by the manufacturer.

**Processing parameters**		**Mechanical properties (dry)**	**Standard**	**XY**	**XZ**	**YZ**
Nozzle temperature	260 - 280 °C	Print direction		Flat	On its edge	Upright
Bed temperature	100 - 120 °C	Tensile strength	ISO 527	103.2 MPa	–	18.2 MPa
Bed material	PEI or glass	Elongation at break	ISO 527	1.80 %	–	0.50 %
Nozzle diameter	≥0.6 mm, Ruby	Young's modulus	ISO 527	8386 MPa	–	3532 MPa
Print speed	30–80 mm/s	Flexural strength	ISO 178	160.7 MPa	171.8 MPa	50.8 MPa
		Flexural modulus	ISO 178	8258 MPa	7669 MPa	2715 MPa
**General properties**		Flexural strain at break	ISO 178	2.40 %	2.80 %	1.80 %
Print part density (dry)	1232 kg/m3	Impact strength Charpy (notched)	ISO 179-2	4.8 kJ/m^2^	3.9 kJ/m^2^	1.3 kJ/m^2^
		Impact strength Charpy (unnotched)	ISO 179-2	20.6 kJ/m^2^	19.3 kJ/m^2^	2.9 kJ/m^2^
**Thermal properties**		Impact Strength Izod (notched)	ISO 180	4.9 kJ/m^2^	5.1 kJ/m^2^	–
HDT at 1.8 MPa (dry)	92 °C	Impact Strength Izod (unnotched)	ISO 180	16.4 kJ/m^2^	18.1 kJ/m^2^	2.9 kJ/m^2^
HDT at 0.45 MPa (dry)	145 °C					
Glass transition temperature	70 °C					
Crystallization temperature	180 °C					
Melting temperature	234 °C					
Melt volume flow rate	145 °C					

The process parameters are shown in Table 3. Each build cycle is carried out with the same process parameters except infill parameters which depend on the cases analysed in section 2.4.

**Table 3:** Parameters of Ultimaker S5 AM machine. Infill parameters change between different cases, the rest remain unchanged.

**Quality**		**Infill (100% case)**	
Layer Height	0.2 mm	Infill Density	100 %
Initial Layer Height	0.4 mm	Infill Pattern	concentric
Line Width	0.58 mm		
		**Material**	
**Walls**		Build Volume Temperature	30 °C
Wall Thickness	1.74 mm	Printing Temperature	265 °C
Wall Line Count	3	Build Plate Temperature	85 °C
		Build Plate Temperature Initial Layer	90 °C
**Top/Bottom**			
Top/Bottom Thickness	1.2 mm	**Speed**	
Top Thickness	1.2 mm	Print Speed	45 mm/s
Top Layers	6	Print Acceleration	4000 mm/s^2^
Bottom Thickness	1.2 mm		
Bottom Layers	6	**Cooling**	
Initial Bottom Layers	6	Enable Print Cooling	Yes
Top/Bottom Pattern	Concentric	Fan Speed	1 %
Bottom Pattern Initial Layer	Concentric		
**Infill (20 % case)**			
Infill Density	20 %		
Infill Pattern	Triangles		

### 2.4 Infill

AM inserts offer the flexibility of a broad range of infill options. While solid inserts are capable of withstanding higher loads [26], the trade-off lies in the increased build cycle time and cost due to the greater volume of material deposited. Given the comparative focus of this experiment, a comparison is drawn between inserts with 20% infill and those with 100% infill. This comparison aims to balance the structural integrity of the inserts with the efficiency of the manufacturing process.

Additive manufacturing (AM) materials exhibit optimal mechanical properties when the direction of internal stresses is perpendicular to the stacking direction. As demonstrated in Section 2.1, the stress distributions in the specimens' critical sections are oriented in the normal direction. A circumferential pattern can ensure this alignment, making it the most suitable pattern to withstand the anticipated flexo-tensile stresses in the insert's critical sections. Consequently, this pattern is employed in the perimetral areas. For inserts with a 20% infill, this pattern is combined with commonly used triangular patterns in the interior areas. Conversely, for inserts with a 100% infill, the entire structure is printed using a circumferential pattern. Reference inserts of CF-SMC are manufactured with 100% infill in every specimen due to the nature of its manufacturing process.

### 2.5 Bonding surfaces

The utilization of MEX AM machines in the fabrication of the AM inserts enables the incorporation of macroscopic surface patterns. These patterns modify the mechanical properties of the bond between the CFRP and the AM material. An increase in surface energy, correlating with an expanded contact surface area, facilitates the formation of stronger adhesive bonds [31]. Furthermore, mechanical interlock potentially improves with macroscopic patterns.

**Fig. 10:**
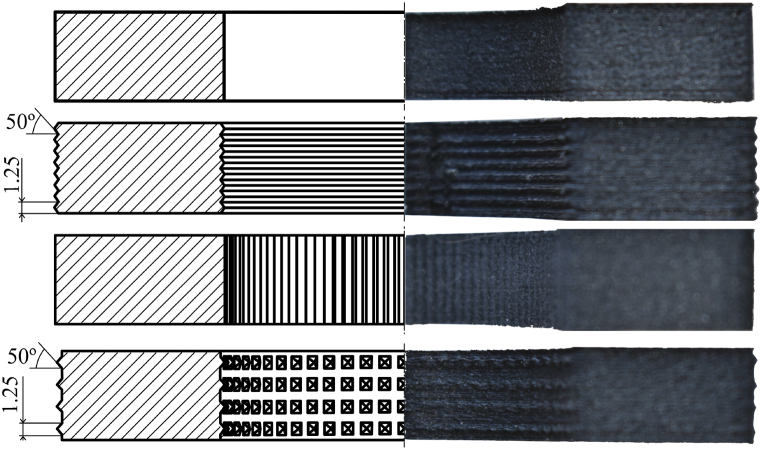
On the left, schematic representation of the different surface geometries designed. From top to bottom: smooth surface, circumferential corrugation, axial corrugation and pyramid-shaped protrusions. Axial corrugation pattern dimensions coincide with circumferential corrugation pattern. On the right, half cut of the geometries.

The incorporation of macroscopic roughness, exemplified by grooves on the millimetre scale, serves to impede the propagation of interfacial cracks. It is observed that the mechanical bond strength is inversely proportional to the spacing of the grooves. The groove's orientation relative to the direction of crack propagation significantly influences interface toughness, with a perpendicular orientation being more effective than a parallel [29,30]. Another compelling approach involves the introduction of pin patterns [27,28]. These patterns are generally introduced through additional manufacturing operations, but polymer AM printers can replicate similar patterns that enhance the mechanical interlock. Based on the identified studies addressing macroscopic patterns to enhance adhesive properties, four different surface geometries are designed, as shown in Fig. 10.

Smooth surface, represented at the top of Fig. 10 is the simplest possible design. The insert surface is manufactured without any pattern. The second surface geometry is circumferential corrugation which should impede interfacial crack propagation and improve the mechanical interlock [29,30]. The width of each pattern is determined through a resolution test to obtain the minimum printable size with acceptable resolution, which is 1.25 mm. The semi-angle of the crest is set at 50°, as a compromise between optimising the adhesive bond and manufacturing limitations [31,36]. The introduction of support material to reduce the semi-angle values is discarded in order to avoid post-processing which could affect the bonding performance and, hence, hide the effect of the surface pattern itself [37]. The third surface geometry is axial corrugation in a similar fashion to the circumferentially corrugated pattern. The fourth surface geometry features pyramid-shaped protrusions. These patterns emulate the introduction of pin patterns observed in other studies [27,28]. The designed patterns have the same manufacturing limitations discussed in the other geometries, since these protrusions are directly fabricated in the same printing operation as the inserts. The characteristic dimensions used in the groove patterns are also applied here.

### 2.6 Surface treatment

The adhesive bond strength between the AM insert and the CFRP laminates could potentially be amplified through surface treatments, thereby benefitting the mechanical interlock [13]. The application of a sandblasting treatment on the insert's surface results in the formation of cavities, pores, and irregularities, which facilitate resin penetration [38]. Consequently, inserts both with and without the application of sandblasting treatments are fabricated. This is done to determine whether the observed variation in mechanical properties sufficiently justifies the incorporation of an additional manufacturing process.

### 2.7 Curing process

The curing process of the epoxy resin in CFRP can be executed through a variety of methods. The fabrication of actual CFRP structures is typically conducted using bladder moulding or reverse vacuum bags, either in an oven or a high-pressure autoclave, achieving pressures between 1 and 8 bar. In the production of automobile parts with CF-SMC, alterations in the pressure applied during curing yield similar outcomes, as its impact is obscured by the high variability inherent in the manufacturing process [9]. However, the introduction of AM inserts could potentially mitigate this variability. Thus, it becomes imperative to investigate the disparity in mechanical properties depending on the curing pressure applied to AM inserts. For the creation of specimens, two distinct methods have been chosen to compare the effects of applying low versus high pressures.

**Fig. 11:**
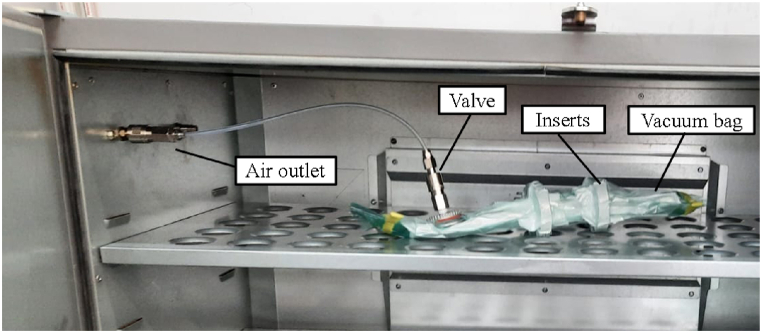
Specimens subjected to a curing process with vacuum bags inside an oven. Two specimens are introduced inside each vacuum bag. Peel ply and breather fabrics are allocated inside. The air is removed by an air pump through the air outlet located on the left part of the oven.

The chosen method for low-pressure curing is executed using vacuum bags inside an industrial oven. The laminated inserts are encapsulated within nylon bags, which are subsequently positioned in an oven set at a temperature of 125 °C for a duration of 30 min. Concurrently, a vacuum pump is employed to evacuate the air from the bags, thereby exerting a pressure of approximately 1 bar onto the specimen's surface. These specific pressure and temperature parameters are calibrated to ensure that the AM insert does not surpass the HDT. Fig. 11 provides an example of the vacuum bag curing process in action.

**Fig. 12:**
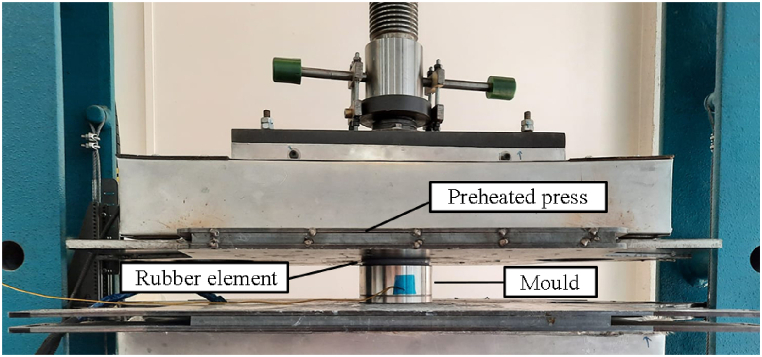
Specimen subjected to thermal press curing. The specimen is located inside the aluminium mould. A preheated press compresses it through a rubber element.

The chosen method for high-pressure curing is executed using thermal press curing. This method simulates the pressure and temperature conditions of an autoclave, but with the added advantages of reduced costs and cycle times [39]. The specimen is placed into an aluminium mould that can rapidly heat up. The mould is heated to 125 °C, and a press applies pressure via a rubber element to the flat face of the insert for a duration of 30 min. Owing to the Poisson effect, the insert deforms in directions perpendicular to the applied force, thereby exerting pressure on the epoxy resin against the mould wall. The pressure that the flat faces of the inserts can withstand is set at a value such that the HDT is exceeded, resulting in the deformation of the insert. As per the manufacturer's specifications, the HDT of PAHT CF15 at 4.5 bar is 128 °C, and at 18 bar is 91 °C. However, the pressure is experimentally determined by pressing inserts without CFRP laminates and visually assessing if the inserts deform. The pressure on the flat face, which ensures deformation in every insert pressed at 125 °C, is found to be 64 bar. Consequently, thermal press curing is conducted at 64 bar, and the effects of applying a higher pressure that exceeds the HDT are compared with the vacuum bag method. Fig. 12 illustrates the execution of the thermal press curing process.

Reference specimens of CF-SMC undergo curing exclusively via thermal press curing. When vacuum bags are utilized, the maximum pressure that can be exerted in an oven is limited to 1 bar. In contrast, the curing processes typically employed in the fabrication of actual structures achieve pressures ranging between 6 and 8 bar. Consequently, the production of specimens through vacuum bagging is not considered.

### 2.8 Ring tensile test

**Fig. 13:**
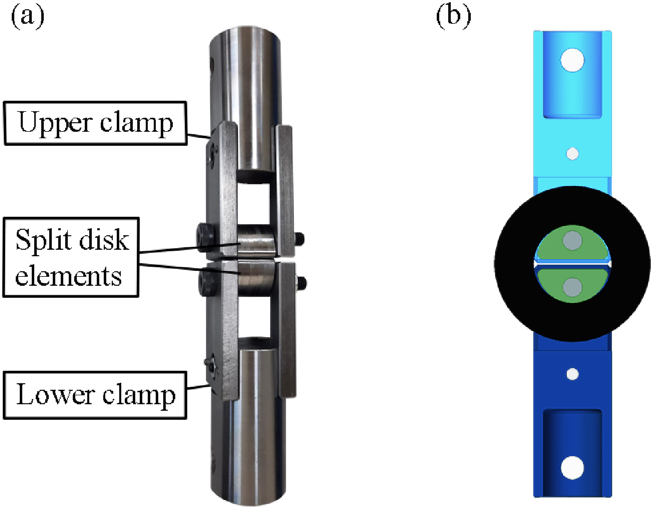
(a) Manufactured tooling. Both lower and upper clamps are assembled similarly with one cylindrical part and two flat parts. The split disk elements connect the clamps to the specimen. (b) Cut section of the tooling with the specimen. Dark blue stands for the lower clamp, light blue for the upper clamp, green for split disks elements, grey for union elements and black for the specimen.

Once the load case affecting the bearing seat structures is understood and the specimens are defined, a tensile test replicating the load distribution shown in Fig. 13 must be developed. The ASTM D 2290 standard, which is frequently referenced in similar studies [[40], [41], [42]], is suitable for this scenario. The tooling proposed in the standard comprises two split disks that are free to rotate around an axis perpendicular to the specimen's plane, enabling them to align with the direction of the applied test force in a universal testing machine [43]. Fig. 13 illustrates both the designed and the actual manufactured tooling, which consists of.

1) Lower clamp: bonds the tooling to the base of the machine and emulates the fixed supports.
2) Upper clamp: bonds the tooling to the movable crossbar and emulates the load distribution.
3) Split disk elements: transfer the load distribution from the clamps to the specimen.

The tests are conducted using an Ibertest ELIB-30-W universal testing machine, which allows both load-controlled and displacement-controlled tests. According to other studies [44,45], both approaches differ fundamentally in the data acquired in the region of the load-displacement curve after the peak point is reached, being the displacement-controlled method more suitable to obtain accurate data in this situation when the specimens show a ductile behaviour [31,32]. However, the materials used for manufacturing the specimens, analysed in section 2.3 show a brittle behaviour. Therefore, the specimens rupture coincides with the peak point of the load-displacement curve, so both control methods are suitable in this experiment.

In the present work, the load-controlled method is preferred for two reasons. Firstly, given that the key parameter of interest in comparing the specimens is their ultimate strength, the load-controlled method is more suitable for determining the force value at the break point. Secondly, the materials used to manufacture the specimens are characterized by high stiffness and fragility. Consequently, in load-controlled tests, displacement values tend to increment gradually with the progression of the load. In contrast, in displacement-controlled tests, load values tend to escalate abruptly as the displacement increases. Therefore, load-controlled tests provide superior resolution for acquiring precise data prior to the specimens’ rupture. The testing speed is established at 0.05 kN/s.

**Fig. 14:**
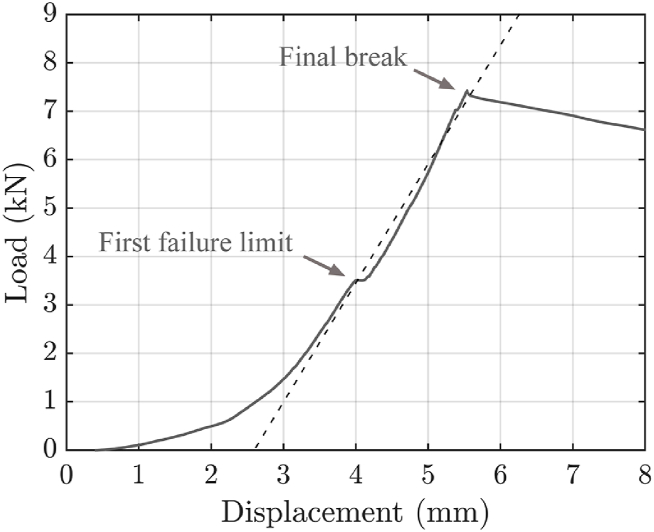
Example of a load-displacement curve obtained in the ring tensile test designed. A first failure limit appears in the middle of the curve, whereas the final rupture occurs without plastic deformation. Dotted line represents the linear approximation employed for stiffness calculations.

The expected load-displacement curve for this test is depicted in Fig. 14. During testing, an initial non-linear segment is observed. This behaviour is usually attributed to equipment adjustments or initial material settling [46]. Once this initial segment is overpassed, a constant stiffness behaviour is observed. This stiffness is compared across different specimens. Potential discontinuities are observed midway through the experiment, usually attributed to local delamination of the adhesive joint or the rupture of the internal structure of the CFRP laminates [9]. Despite initial failures, no substantial change in stiffness is observed. The test proceeds until the complete fracture of the specimen, which typically occurs in the sections farthest from the points of tensile force application, as justified in section 2.1. Given that none of the materials used to fabricate the specimens undergo plastic deformation, their tensile strength and ultimate strength are expected to match.

### 2.9 Test plan

The objective of the test is to draw a comparison between the AM inserts and the reference specimens. To execute this comparative analysis, several key parameters will be considered. These include the magnitude of the force that induces the initial failure, the breaking limit, and the stiffness of the specimens.

AM inserts can be manufactured by combining the different design and manufacturing parameters explained in sections 2.4 through 2.7. Given the substantial number of potential combinations of the parameters, a test plan to reduce the number of tests, and thus the number of specimens to manufacture, is developed. This test plan facilitates the identification of the optimal parameter combination. This selected combination is then compared with the reference specimens. Table 4 shows all the potential variable combinations.

**Table 4:**
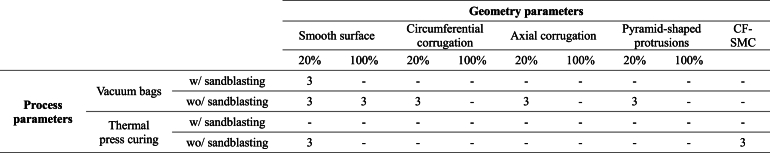
Summary of the combination of parameters in the tested specimens. The quantity of specimens manufactured of each combination is specified.

During the initial phase, the influence of surface geometry is studied. Inserts representing each surface geometry are subjected to testing. These inserts are fabricated without the application of sandblasting treatment and with a 20% infill, primarily due to their expedited and cost-efficient manufacturing process. The curing of the resin is conducted using vacuum bags because 20% infill inserts would not withstand the high pressure levels suffered during thermal press curing.

In the subsequent phase, the impact of the sandblasting treatment is examined. Specimens which were identified in the initial phase as having the most efficacious surface geometry are fabricated. These specimens maintain the same infill and curing process parameters. However, the contact surface of the insert, which interfaces with the prepreg layers, is subjected to a sandblasting process.

In the next phase, the influence of infill is investigated. Specimens which exhibit the optimal combination of geometry and surface treatment as determined in the second phase are fabricated using the vacuum bag curing method. However, these specimens are produced with 100% infill inserts for comparison against the 20% infill inserts. While it is anticipated that the 100% infill inserts will deliver superior results [26], it remains to be ascertained whether this difference justifies the weight increment.

In the following phase, the impact of compaction pressure is examined. Inserts, which represent the optimal combination of geometry and surface treatment as determined in the third phase, are fabricated. However, the distinguishing factor here is that the resin undergoes curing via thermal press curing. This method applies a higher degree of compaction pressure compared to vacuum bag curing. Consequently, specimens with 100% infill are utilized to guarantee their ability to endure the compaction. This approach facilitates a comparative analysis of the results when there is a variation in the compaction pressure.

In the final phase, the best combination of the previous parameters is compared with the reference inserts made of CF-SMC.

The manual lamination process of the prepreg sheets introduces a high degree of variability in the manufacturing process. To bolster the reliability of the results, three specimens are fabricated for each tested parameter combination as specified in Table 4. This approach allows the calculation of both the mean and variability of the results for each combination.

## 3 Results and discussion

### 3.1 Experimental results

**Fig. 15:**
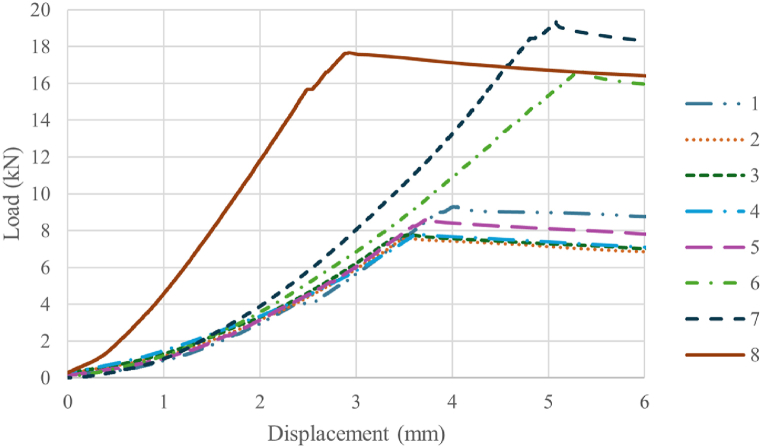
Typical load-displacement curves for each type of specimens. Test numbers are listed in Table 5.

**Fig. 16:**
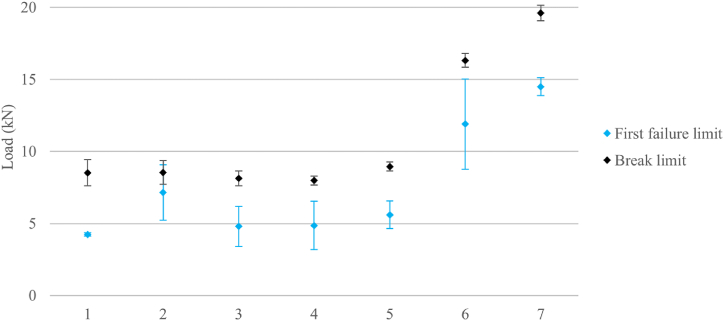
Differences in mean and confidence interval of first failure limits and break limits of all AM inserts tested. IDs as listed in Table 5.

The conducted tests are carried out following the test plan detailed in section 2.9. Table 5 provides a summary of the experimental results. This table includes the mean values and standard errors for each tested parameter combination, offering a snapshot of the data for easy interpretation and comparison. Typical load-displacement curves, observed during mechanical testing for each specimen type, are shown in Fig. 15. No significant difference in stiffness is observed among AM core specimens. Consequently, the study of the effect of the various design and manufacturing parameters for such specimens focuses on fracture limit and first failure limit. The results corresponding to the specimens manufactured with AM cores, denoted by IDs 1 to 7 in the table, are also visually represented in Fig. 16.

**Table 5:** Means and standard errors of first failure limit and break limit of the different combination of parameters in AM inserts and the reference inserts of CF-SMC.

ID	Surface geometry	Surface treatment	Internal structure	Curing process	First failure limit mean and standard error (kN)	Break limit mean and standard error (kN)	Stiffness mean and error (kN/mm)
1	Smooth Surface	Wo/ sandblasting	20% infill	Vacuum bags	4.25 ± 0.11	8.52 ± 0.33	2.626 ± 0.035
2	Circumferential corrugation	Wo/ sandblasting	20% infill	Vacuum bags	7.15 ± 1.92	8.54 ± 0.91	2.694 ± 0.182
3	Axial corrugation	Wo/ sandblasting	20% infill	Vacuum bags	4.80 ± 1.39	8.13 ± 0.83	2.391 ± 0.254
4	Pyramid-shaped	Wo/ sandblasting	20% infill	Vacuum bags	4.87 ± 1.67	7.99 ± 0.52	2.180 ± 0.264
5	Smooth Surface	W/ sandblasting	20% infill	Vacuum bags	5.60 ± 0.96	8.95 ± 0.31	2.428 ± 0.196
6	Smooth Surface	Wo/ sandblasting	100% infill	Vacuum bags	11.90 ± 3.12	16.32 ± 0.48	3.686 ± 0.119
7	Smooth Surface	Wo/ sandblasting	100% infill	Thermal press	14.51 ± 0.61	19.61 ± 0.54	4.540 ± 0.199
8	CF-SMC			Thermal press	12.33 ± 4.02	18.05 ± 0.71	6.228 ± 0.613

The initial phase of the experiment is designed to evaluate the performance of various surface geometries. The results, as depicted by IDs 1 to 4, reveal that specimens with a circumferential corrugation surface geometry exhibit a first failure limit that is 68% higher than that of specimens with a smooth surface. Specimens with axial corrugation and pyramid-shaped protrusions demonstrate first failure limits that fall within the same confidence intervals as those with smooth surfaces and circumferential corrugations. Despite the differences in surface geometry, the break limits are similar across all specimens, as indicated by the overlapping confidence intervals. Similar tests with specimens of CFRP laminates bonded to surfaces with grooves patterns also exhibit superior mechanical behaviour, however, they show a higher dispersion in results compared to the smooth specimens [29]. It is concluded that the smooth surface specimens are the preferred choice, despite having a lower first failure limit than the circumferential corrugation specimens. This preference is due to their lower result variability, which is advantageous for the final comparison with CF-SMC specimens, known for their high variability in the manufacturing process.

The second phase of the experiment is conducted to evaluate the effectiveness of sandblasting surface treatment. The results, as shown by IDs 1 and 5, reveal that the first failure limit in specimens subjected to sandblasting is 32% higher than in those without this post-treatment. While the break limits of both types of specimens are similar due to overlapping confidence intervals, there is a greater dispersion in specimens that did not undergo sandblasting. These findings, particularly those related to fracture limit values, seem to contradict the observations made by Bo Tan et al. [38]. However, it's important to note that in their study, the authors focus solely on the shear strength of the bond. In contrast, in this work several potential failure mechanisms are possible, as explained in Section 2.1. The lack of a significant impact of sandblasting on the overall strength of the specimen could be attributed to the fact that failure is due to either a core break or a CFRP break, rather than a bonding failure.

The third phase of the experiment aims to evaluate the impact of different infill percentages. IDs 1 and 6 show the results of this comparison. The first failure limit for specimens with 100% infill is 180% higher than those with 20% infill. Additionally, the break limit improves by 92%. These findings align with studies that investigate the influence of infill on individual MEX/P parts, which report an increase in both strength and stiffness as the infill percentage rises [26]. The increase in the overall break limit of the specimen due to strength increase of the AM core is obvious. The effect of the increase in stiffness, however, warrants further explanation. An increase in stiffness mitigates deformations and, thereby diminishing both shear loads on the bonding surface and tensile loads in the CFRP, resulting in a higher overall fracture limit. An unexpected finding in 100% infill specimens is the high variability in first failure limit despite the fact that the selected parameter combination was intended to minimize variability. This discrepancy is likely due to the significant sources of variability inherent in the curing process of the CFRP [47].

The fourth phase of the experiment is designed to evaluate the efficacy of various curing processes. IDs 6 and 7 present the results of this comparative analysis. The data shows that the initial failure limit for specimens produced using vacuum bags and thermal press curing is comparable, as indicated by the overlap in their confidence intervals. However, the results exhibit significantly less variability with thermal press curing. This finding suggests that an increase in infill percentage does not necessarily alter the variability of the results. The increased variability observed in the third phase can be attributed to the inherent variability in the manufacturing processes [47]. The break limit of thermal press curing specimens is 20% higher than that for specimens cured with vacuum bags, which indicates that the compaction suffered by the insert with higher pressures increases its strength. In the case of CF-SMC parts, discerning the impact of changes in curing pressure on mechanical properties is not possible due to the natural variability of the material masking these effects [9]. However, for AM inserts, higher curing pressures are demonstrated to augment the strength of the specimen.

**Fig. 17:**
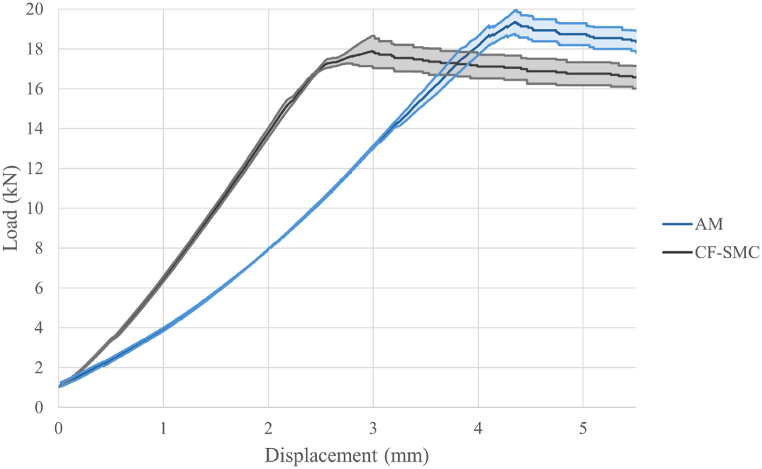
Mean and confidence regions of AM specimens (ID 7) and CF-SMC specimens (ID 8).

**Fig. 18:**
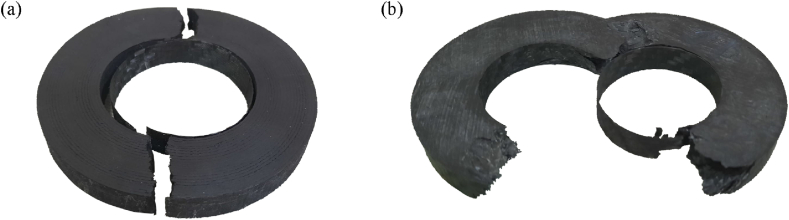
(a) AM core specimen after ring tensile test. (b) CF-SMC specimen after ring tensile test.

The fifth phase of the experiment was designed to compare the properties of the most performant AM core specimens with those of CF-SMC specimens. Fig. 17 illustrates the average load-displacement curves and their confidence regions. From the data, two salient outcomes emerge. First, the AM core specimens (referenced as ID 7 in Table 5) show a break limit that is 9% higher than that of the CF-SMC specimens. Second, they demonstrate a 27% decrease in stiffness compared to CF-SMC. Given that high stiffness is a characteristic typically associated with carbon fibre composites, the reduced stiffness exhibited by the AM core specimens aligns with expectations. However, this reduction should not necessarily be considered as a disadvantage. The extremely high stiffness of CFRPs usually leads to brittle sudden fracture of the structures. Typical fractures observed in Fig. 18 support this theory. Despite both fractures occurring instantaneously during testing, AM specimens show a more predictable fracture surface where the crack propagates through the critical section. CF-SCM specimens, however, show an irregular fracture surface, suggesting that the fracture is probably initiated at multiple points as it usually occurs in brittle material. The combination of lower stiffness and a higher fracture limit could potentially enhance the strength of the structure while also providing a more gradual and predictable fracture mechanism. Furthermore, AM core specimens demonstrated greater result stability compared to CF-SMC specimens, as evidenced in Table 5. This stability could be an additional advantage in certain applications.

### 3.2 Analytical results

The mathematical model delineated in section 2.1 provides insights into the stress distributions within critical sections of AM specimens. This model explains the underlying cause of the ultimate failure of AM specimens, as it allows for a comparison of stress values with the breaking limits of materials. The materials employed in section 2.3 and the geometric dimensions detailed in section 2.2 are taken into account when considering the final rupture limit of AM specimens during the fifth phase, represented in Fig. 17. The mathematical model results are presented in Table 6.

**Table 6:** Stresses obtained in critical operation points at final break of AM specimens in the fifth phase of the experiment. These values are obtained through the structural model developed in section 2.1.

**AM material**	σ_core_ (-10mm)	103.9 MPa
	σ_core_ (10 mm)	−24MPa
**CFRP**	σ_CFRPin_	669 MPa
	σ_CFRPOut_	−132MPa
**Bonding interface**	τ_in_	565 MPa
	τ_out_	108 MPa

Observations reveal that the inner portion of the critical section is subjected to tensile loads due to both tensile and flexural components. Conversely, the outer part exhibits a net compression stress state, resulting from the summation of tensile and flexural components. As predicted by the analytical model, the fracture initiates in the inner part of the critical section. However, it remains indeterminable whether the initial fracture occurs in the AM core or in the CFRP, as both materials approach their ultimate strength. It can be concluded, though, that the failure does not occur in the bonding interface.

The congruence between the analytical model and the experimental results validates the model as a useful tool for predicting the behaviour of various material combinations and ring geometries. Although it does not entirely represent the case study of bearing seats, it serves as a simplified model, provided its limitations are considered. These limitations include not taking surface variables into consideration, considering only 100% infill solid isotropic cores, and accounting solely for radial bearing loads.

### 3.3 Economic considerations

This paper has proved that additive manufacturing (AM) cores present a viable alternative to carbon fibre sheet moulding compound (CF-SMC) for bearing seat structures. The technical advantages and disadvantages of AM cores may vary depending on the specific application. However, the economic benefits of AM cores, which are universally applicable, warrant consideration.

AM cores hold two substantial advantages over CF-SMC. Primarily, the fabrication of AM cores is an automated process, contrasting with the often manual stacking involved in CF-SMC processes, thereby reducing labour costs. This automation not only reduces direct labour costs but also minimizes the potential for human error, further enhancing cost-effectiveness. Moreover, the automated nature of AM core production allows for easy scaling of operations. As demand increases, production can be ramped up without a significant increase in labour costs.

Secondly, the repeatability of AM cores surpasses that of CF-SMC, resulting in decreased quality costs associated with defective units. The higher repeatability rate means units of the same quality and specifications can consistently be produced, leading to a reduction in the number of defective units produced, thereby decreasing the costs associated with quality control, rework, and waste.

## 4 Conclusions

This paper successfully demonstrates the feasibility of using additive manufacturing (AM) inserts as cores for carbon fibre reinforced polymer (CFRP) structures to withstand out-of-plane and flexural loads. This innovative approach serves as a viable alternative to the current state-of-the-art carbon fibre sheet moulding compound (CF-SMC) structures. The case study focuses on bearing seat structures, and the results are promising, with improvements of 17.7% in the first failure limit and 8.6% in the break limit. Furthermore, more stable values are observed with lower standard error values.

A ring tensile test methodology is developed to analyse the influence of different design and manufacturing parameters. The variables studied include surface geometry, surface treatment, internal structure, and curing process. Surface geometry and surface treatment do not show significant effects on the mechanical behaviour of specimens. However, the internal structure shows a 190% increase in the break limit when changing from 20% infill to 100% infill. The curing process also has a high influence, with thermal press curing (a high-pressure process) showing a break limit 20% higher than vacuum bag curing (a low-pressure process).

A simplified analytical model is developed to predict the strength and potential failure mechanisms of the specimens. This model is a valuable tool for future design decisions as it allows for sizing the structure based on the required loads. However, the model has some limitations, as it only considers 100% infill cores, it does not consider the surface variables, and it only considers radial bearing loads.

From an economic perspective, AM cores have two significant advantages over CF-SMC. Firstly, the production of AM cores is an automated process, whereas CF-SMC processes are often stacked manually, leading to lower hand-labour costs. Secondly, the repeatability of AM cores is higher, leading to lower quality costs due to faulty units.

It can be concluded that additive manufacturing (AM) inserts have the potential to serve as reinforcements for bearing seats in carbon fibre reinforced polymer (CFRP) structures. The implementation of AM inserts could enhance stability and strength. However, it is essential to clarify that this study does not assure the successful operation of these inserts in real-world applications; rather, it demonstrates the possibility of testing full structures with such integrated inserts. The adjustment of bearings with these structures warrants further investigation, given the challenges caused by the low resolution of material extrusion of polymers (MEX/P). To ascertain their practical applicability, additional research and validation in real-world bearing seat structures are indispensable.

## Ethics declarations

Review and approval by an ethics committee were not needed for this study because this study did not involve animal or human experiments.

## Data availability

All the relevant data are included in the manuscript. No separate repository is attached.

## AI disclosure statement

During the preparation of this work the authors used Microsoft Copilot in order to improve language and readability. After using this tool, the authors reviewed and edited the content as needed and take full responsibility for the content of the publication.

## CRediT authorship contribution statement

**Guillermo Retuerta del Rey:** Writing – review & editing, Validation, Supervision, Project administration, Methodology, Investigation, Formal analysis, Data curation, Conceptualization. **Javier de Lucas Salgado:** Writing – review & editing, Writing – original draft, Visualization, Methodology, Investigation, Data curation. **Alex Alberto González Hernández:** Investigation. **Enrique Chacón Tanarro:** Writing – review & editing, Supervision, Project administration, Funding acquisition.

## Declaration of competing interest

The authors declare that they have no known competing financial interests or personal relationships that could have appeared to influence the work reported in this paper.

## Abbreviations

A: Section studied in energy term
A: Section in half of insert representation in negative x axis
A+: Section in half of insert representation in positive x axis
AM: Additive manufacturing
b: Length of the section
B: Section in half of insert representation in y axis
CF-SMC: Carbon Fibre Sheet Moulding Compounds
CFRP: Carbon Fibre Reinforced Polymer
E: Young's modulus
E_CFRP_: Young's modulus of the CFRP laminates
E_Core_: Young's modulus of the core material
f_1_: Bearing load distribution per area unit
f_max_: Maximum value of the load distribution per area unit
F_t_: Resultant of the force applied along the y axis
HDT: Heat Deflection Temperature
I_z_: Moment of inertia in z axis
k: Constant term of flexural stress distribution
l: Thickness of the insert along the z axis
M_b_: Flexural momentum supported by section B
MEX/P: Material Extrusion of Polymers
M_f_: Flexural momentum in a section of the specimen
M_z_: Flexural modulous in section A
M_z+_: Flexural modulous in section A+
N: Normal reaction force
PA: Polyamide
q: Constant term of tensile stress distribution
r_0_: Guideline radius of the ring specimen
r_1_: Inner radius of the ring specimen
r_2_: Outer radius of the ring specimen
R_in_: Total load in the inner laminate
R_out_: Total load in the outer laminate
R_xb_: Reaction force in x axis in section B
R_y_: Reaction force in y axis in section A
R_y+_: Reaction force in y axis in section A+
t: Laminates thickness
U_Mb_: Energy of the model due to Mb
ur: Unitary vector in radial direction
ux: Unitary vector in x axis
uy: Unitary vector in y axis
uz: Unitary vector in z axis
X: X axis
Y: Y axis
Z: Z axis
θ: Angle of study of the inset from the x axis
σ_CFRPin_: Tensile stress in the inner laminate
σ_CFRPout_: Tensile stress in the outer laminate
σ_Core_: Total stress distribution in the core
σf: Flexure stress distribution in the core
σ_n_: Tensile stress distribution in the core
τ_in_: Shear stresses developed in the bond at the inner laminate
τ_out_: Shear stresses developed in the bond at the outer laminate
